# Effects of Feeding Level and Breed Composition on Intake, Digestibility, and Methane Emissions of Dairy Heifers

**DOI:** 10.3390/ani11030586

**Published:** 2021-02-24

**Authors:** Tainá Silvestre, Marina A. Lima, Gustavo B. dos Santos, Luiz G. R. Pereira, Fernanda S. Machado, Thierry R. Tomich, Mariana M. Campos, Arjan Jonker, Paulo H. M. Rodrigues, Virginia L. N. Brandao, Marcos I. Marcondes

**Affiliations:** 1 College of Veterinary Medicine and Animal Science, Universidade de São Paulo, Pirassununga 13635–900, São Paulo, Brazil; tainasilvestre@usp.br (T.S.); pmazza@usp.br (P.H.M.R.); 2 Department of Animal Sciences, Universidade Federal de Viçosa, Viçosa 36570–900, Minas Gerais, Brazil; marinalima17@hotmail.com; 3 Faculty of Veterinary, Universidade Federal Fluminense, Niterói 24220–900, Rio de Janeiro, Brazil; gberviansantos@hotmail.com; 4 Brazilian Agricultural Research Corporation, Embrapa Gado de Leite, Juiz de Fora 36038–330, Minas Gerais, Brazil; luiz.gustavo@embrapa.br (L.G.R.P.); fernanda.machado@embrapa.br (F.S.M.); thierry.tomich@embrapa.br (T.R.T.); mariana.campos@embrapa.br (M.M.C.); 5 Grasslands Research Centre, AgResearch Ltd., Palmerston North 4442, New Zealand; arjan.jonker@agresearch.co.nz; 6 Micronutrients USA LCC, Indianapolis, IN 46231, USA; virginia.brandao@micro.net; 7 Department of Animal Sciences, Washington State University, Pullman, WA 99163, USA

**Keywords:** crossbred Holstein × Gyr, dairy cattle nutrition, feed efficiency, greenhouse gas, Holstein, Gyr

## Abstract

**Simple Summary:**

The nutrients requirements of dairy cattle is the most used system to formulate dairy cattle diets. However, these guidelines were developed based on research using mostly purebred Holstein in temperate conditions and did not determine nutrient requirements of crossbred Holstein × Gyr or Gyr animals raised in a tropical environment. Studies conducted in tropical conditions demonstrated that breed largely affected the nutrient requirements of crossbred and Gyr cattle, which limits the use of this system. Our objective was to evaluate the effects of two feeding levels and three breed compositions on nutrient intake, digestibility, performance, and methane emissions of prepubertal (10-month-old) dairy heifers. We observed that feeding diets to allow gains of 400 and 800 g/day resulted in greater daily gain than the formulated, overestimating the nutritional requirements of the heifers. Overall, breed composition did not affect dry matter intake, but it did result in differences in nutrients digestibility coefficients and feed efficiency. Feeding prepubertal heifers above maintenance requirements was the main driver in reducing CH_4_ intensity in confinement conditions.

**Abstract:**

The dairy Nutrients Requirements of Cattle (NRC) was developed using data from purebred Holsteins and it might not accurately predict the performance of crossbred cattle. Our objectives were to evaluate the effects of two feeding levels (FLs) and three breed compositions (BCs) on nutrient intake, digestibility, performance, and methane (CH_4_) emissions of prepubertal dairy heifers. We used thirty-six heifers from three BCs: purebred Holstein (H), purebred Gyr (G), and F1 Holstein × Gyr (HG). Each BC had 12 animals and the experiment was designed as twelve incomplete three by three Latin squares, in a factorial arrangement three by two, with three BCs and two FLs (400 and 800 g/day). Total tract nutrient digestibility was determined using total fecal collection and DMI was individually measured. The data were analyzed using the PROC MIXED in SAS. Dry matter intake of all nutrients increased from the medium to high feeding level and the nutrients digestibility coefficients did differ among BCs. Achieved body weight gain in the medium FL treatment was greater than those predicted using the NRC, suggesting that crossbred and Gyr heifers have similar performance to Holsteins. Breed composition does not influence body weight gain of confined dairy heifers, but Holstein heifers fed a medium FL had higher feed efficiency and reduced CH_4_ emissions intensity.

## 1. Introduction

The first experiments that focused on breeding Zebu cattle (*Bos indicus*) with Holstein breed (*Bos Taurus*) were published in 1940 and aimed to improve milk production, fertility, and rusticity of dairy animals in tropical countries [1]. This crossbred is now the main basis of the Girolando (Holstein × Gyr) genotype and, due to the good adaptability and milk production, this cross has been widely used in tropical conditions nowadays [2]. Among the main selected traits are milk production and composition, udder health, workability, and their main features are heat stress tolerance and resistance to endo-and ecto-parasites [3].

A recent study using cows with several Holstein × Gyr crosses showed a greater milk yield and shorter dry period length as a result of a higher percentage of Holstein genes in their genetic composition [4]. The results also indicated a high genetic variation of the Girolando breed, which attracts interest from researchers, once production and reproduction can still be greatly improved in breeding programs. Therefore, an intensification of on-farm data collection of this crossbred should be considered to fill this gap regarding intake and utilization of nutrients of Girolando raised in tropical conditions since these cows produce around 80% of the milk in Brazil [4].

The dairy NRC [5] was developed using data from purebred Holstein animals raised in a temperate climate, with little variation in the type of production system and environment conditions. It does not include data from Holstein × Gyr or Gyr animals and it does not account for their body composition. The companion study [6] of the present research indicated that Holstein × Gyr might have greater digestibility of some nutrients than Holstein and highlighted the need for further studies measuring the effect of BC on the digestibility of diet components.

Data comparing the performance of different BCs of Holstein × Gyr is not consistent due to the large influence of production systems, diet composition, and genetic variation found in tropical countries. Studies using animals with standardized genetics, age, and physiological state is warranted [7,8]. Better performance estimates are crucial for dairy operations to achieve adequate and more predictable animal performance, avoiding inefficient use of nutrients and resources. Additionally, effectively meeting animals’ requirements reduces nutrients excretion to the environment and greenhouse gas (GHG) emissions [9]. Therefore, we hypothesize that (1) using NRC [5] recommendations for a target performance will result in different performances of Holstein, Gyr, and crossbred HG heifers; (2) that BC influences the genetic potential to achieve a target body weight gain; (3) Gyr and crossbred HG heifers fed under higher performance levels have greater feed efficiency compared to Holstein heifers. We aimed to evaluate the effects of performance level and breed composition on nutrient intake, digestibility, performance, and methane emissions of prepubertal dairy heifers of three BCs fed at two different feeding levels (FLs).

## 2. Materials and Methods

### 2.1. Ethics Statement

The protocol for the care and manipulation of animals for the accomplishment of this experiment was approved by the Ethics Committee on Animal Use of the University of São Paulo (Protocol no. 3046/2013). The experiment was performed at the Multi-use Livestock Complex on Livestock Bioefficiency and Sustainability at Embrapa Dairy Cattle, Coronel Pacheco, MG, Brazil (21°33′26″ S; 43°15′27″ W) and lasted for 172 consecutive days.

### 2.2. Experimental Design, Treatments, Animals, and Diets

We used thirty-six dairy heifers from three BCs: purebred Holstein, purebred Gyr, and F1 Holstein × Gyr. Each dairy BC had 12 animals with an initial bodyweight (iBW) of 219.8 ± 42.6, 215.8 ± 44.4, and 228.3 ± 44.6 kg for Holstein, Gyr, and F1 ½ Holstein × ½ Gyr, respectively. Heifers were housed in a tie-stall facility with individual feed and water bins. To reduce the variability in the trial, all heifers were contemporaries and 10-months-old on average.

The experiment was designed as twelve incomplete three by three Latin squares, in a factorial arrangement three by two (three BCs and two FLs) and three periods. Each Latin square was composed of one animal of each BC. Within each period, twelve animals (two of each Latin square) were fed to reach two FLs, and the remaining animals were fed at maintenance until the next period. Each experimental period consisted of 42 days of adaptation to the diets, followed by eight days of metabolism measurements as described below. After each experimental period, all heifers went through a 10-day washout period, during which they were permitted *ad libitum* intake of the same experimental diet to allow gain of 400 g/day. This washout period was used to avoid any compensatory weight gain or residual effects in the next periods.

Diets were formulated using NRC (2001) and fed to supply an equal metabolizable energy intake to allow gains of 400 and 800 g/day, corresponding to a medium (MFL) and high feeding level (HFL), respectively. The diet was fed as total mixed ration (TMR), composed of corn silage and concentrate supplement (85:15 w/w based on the dry matter (DM); the chemical compositions of ingredients are presented in Table 1. The TMR was offered once daily at 8:00 am. The BW of each animal was measured every week and used to adjust the amount of feed offered to each heifer.

### 2.3. Dry Matter Intake and Digestibility

Data for the apparent total tract digestibility were taken at three time-points: at the beginning (at 46 days), middle (107 days), and end of the trial (168 days). Each sample collection period lasted eight days: five days of adaptation to the management and three days of total feces collection [10]. The heifers were kept in the tie stall during the digestibility trial. Two people were on duty in shifts of 24 h during the 72 h. We collected the feces as soon as they were produced, using a scoop shovel. Each heifer had one container and immediately after defecating the collected feces were stored inside the container. These procedures prevented both the contamination of feces by urine and the need to keep the heifers in metabolism crates, which would influence the animal behavior. At the end of each sampling day, the feces of each animal were weighed. After homogenization, a sub-sample was taken and frozen for subsequent analysis. One composite sample per animal per period was prepared based on the DM weight for every collection day before proximal analysis. During the fecal sampling events, dry matter intake (DMI) was determined over five consecutive days as DMI, kg/day = feed DM offered, kg/day – feed refusals DM, kg/day. As diets were formulated for a specific metabolizable energy intake, refusals were negligible but recorded and sampled. Representative samples of silage, concentrates, and refusals were collected daily and pooled for chemical analysis. Intake and digestibility data were summarized by period for statistical analysis.

Samples of feeds, refusals, and feces were oven-dried at 55 °C for 72 h, ground through a 1-mm screen (Wiley mill; A. H. Thomas, Philadelphia, PA, USA.), and analyzed for contents of DM (method 930.15), ash (method 924.05), crude protein (CP) (method 984.13), and ether extract (EE) (method 920.39) according to [11]. Neutral detergent fiber (NDF; with heat-stable amylase and sodium sulfite and expressed exclusive of residual ash) and acid detergent fiber (ADF) were analyzed according to [12]. Non-fibrous carbohydrates (NFC) were calculated according to [13]: OM– (NDF_ap_ + CP – CP_u_ + U) + EE), where OM is organic matter, NDF_ap_ is NDF corrected for ash and protein, U is urea, and CP_u_ is CP-derived U. Gross energy (GE) was determined using an adiabatic calorimeter (model C-5000, Labcontrol IKA, São Paulo, SP, Brazil). The metabolizable energy (ME) content was calculated by multiplying the digestible energy by 0.82 [14].

### 2.4. Blood Samples

Blood samples were collected from the coccygeal vein or artery on day three of each digestibility trial, 4 h after feeding. Samples (approximately 10 mL) were collected in vacuumed tubes containing EDTA (and immediately centrifuged at 1400× *g* for 15 min at room temperature. Samples were stored at −20 °C. Non-esterified fatty acids (NEFA) (Randox Laboratories, Ltd., Crumlin, Antrim, UK, Cat # FA 115) concentrations of plasma were determined spectrophotometrically (Shimadzu UV 1601).

### 2.5. Animal Performance

All animals were weighed weekly during the experiment at 8:00 a.m. immediately before feeding. Average daily gain (ADG) during the experimental period was calculated as the linear regression coefficient of live weight. Feed efficiency (FE) was calculated as: FE (kg/kg) = ADG (kg/day)/DMI (kg/day).

### 2.6. Methane Emission Measurement by the Sulfur Hexafluoride Tracer Technique 

The sulfur hexafluoride (SF_6_) tracer technique was used to estimate daily CH_4_ emissions [15] during each digestibility trial. The SF_6_ release rate and expected lifetime of permeation tubes were calculated using the pre-filled weight of SF_6_ within each tube and serial change in weight during an 11-week period within a controlled environment at 39 °C. The release rates of SF_6_ tubes ranged from 1.40 to 1.85 mg/day, with a mean of 1.66 ± 0.147 mg/day, and lifespans of 330 ± 119 days. The permeation tube was orally inserted into the rumen of each heifer using a stomach tube the week before the first measurements started. The breath collections were started 1 h before feeding (7:30 a.m.) over 24 h, repeated on five consecutive days. The expired breath was collected by placing a head collar on each heifer which had a gas collection line integrated that ran from just above the animal’s nostrils to an evacuated canister (-15 PSI). Background concentrations of SF_6_ and CH_4_ were measured daily by hanging two evacuated canisters at either end of the tie-stall barn. Canisters were made of polyvinyl chloride (PVC) equipped with a capillary tube (0.127-mm diameter) that was used to sample the gas with the vacuum inside the canister remaining at 40–60% of the initial vacuum after 24 h of measurement. After 24 h, canisters containing breath samples with SF_6_ and CH_4_ were removed from each heifer, the gas was sampled, and the pressure was recorded. Background canisters were treated in the same way. If the pressure inside the canisters was below or above the 40–60% range, gas samples were not collected, and an additional CH_4_ measurement day was added to ensure that at least five days of breath samples were collected from each animal. Gas samples from each canister (20 mL) were collected into five pre-evacuated 12 mL Exetainers (Labco Ltd., Lampeter, UK). The SF_6_ (ppt) and CH_4_ (ppm) concentrations in the samples were determined using two separate gas chromatographs: Agilent models 6890N plus and 7820A, respectively (Agilent Technologies, Santa Clara, CA). Both chromatographs were equipped with a split-splitless injector, but a μECD detector (electron capture) was used to measure SF_6_ and an FID detector (flame ionization) was used to measure CH_4_ concentration.

For SF_6_ analysis, a 30 m × 0.530 mm × 25.0 μm column (HP-Molsieve, Agilent Technologies, Santa Clara, CA, USA) was used with N_2_ as carrier gas at a flow rate of 5.0 mL/min with N_2_ as the makeup gas at 40 mL. The μECD detector was maintained at 300 °C and N_2_ at 40 mL/min was used as the carrier gas. The oven temperature was kept at 50 °C for 4 min to elute the desired constituents. The gas chromatograph was calibrated weekly using SF_6_ (White Martins, São Cristóvão, RJ, Brazil) standards ranging in concentrations: 30, 100, 500, 1500, 3000 ppt. The CH_4_ concentration was analyzed using two columns, a 30 m × 0.530 mm × 40.0 μm column (HP-Plot/Q, Agilent Technologies, Santa Clara, CA, USA) and a 30 m × 0.530 mm × 25.0 μm column (HP-Molsieve, Agilent Technologies, Santa Clara, CA, USA) with H_2_ as carrier gas at a flow rate of 7.0 mL/min. The FID detector was maintained at 280 °C, 10 mL/min of H_2_ flow, 400 mL/min of synthetic air, and 20 mL/min of complementation flow. The oven temperature was kept at 50 °C for 4.5 min to elute the desired constituents. The gas chromatograph was calibrated using CH_4_ (Linde AG, Rio de Janeiro, RJ, Brazil) at 4.8, 9.7, 19.6, 102, and 203 ppm. The emission rate of enteric CH_4_ (ECH_4_; g/animal/d) was calculated from the measured SF_6_ and CH_4_ concentrations sampled from the canisters ((CH_4_)M; ppm and (SF_6_)M; ppt) minus the background SF_6_ and CH_4_ concentrations ((CH_4_)BG; ppm and (SF_6_)BG; ppt) (converted with the molecular mass of CH_4_ (MW CH_4_ = 16) and SF_6_ (MWSF_6_ = 146)) and multiplied by the predetermined release rate of the permeation tubes (RSF_6_; mg/d) as described by [16]:

ECH_4_ = RSF_6_ × (CH_4_ M − CH_4_ BG)/(SF_6_ M − SF_6_ BG) × (MW CH_4_)/(MW SF_6_) × 100

### 2.7. Statistical Analysis

The data were analyzed by analysis of variance, using the mixed model procedure (PROC MIXED), with BCs (Holstein, Gyr, and F1 Holstein × Gyr), FLs (400 and 800 g/day), and their interaction as fixed effects, and animal nested within square, squares and periods as random effects. Initial BW was included as a covariate in all analyses and was removed from the model when the covariate was non-significant. For all significant responses, the Student’s *t*-test was used to identify differences among least squared means. All analyses were performed using PROC GLIMMIX of SAS University Edition, considering statistical differences when *p* < 0.05 and trends when 0.05 < *p* < 0.10.

## 3. Results

There was a FL effect on intake of all nutrients (Table 2), increasing from the medium to high feeding level (*p* < 0.001). There was no interaction between FL and BC.

The BC, FL, and their interaction were not significant (*p* > 0.05) for any of the digestibility parameters, except for CP (Table 3). Gyr heifers had CP digestibility coefficient similar to crossbred HG heifers and these values were 3.6% higher than those of Holstein heifers.

**Figure 1:**
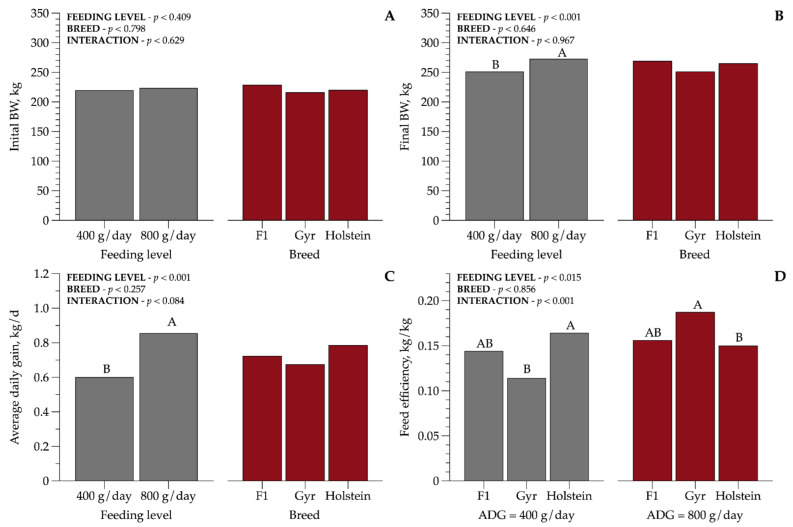
Initial and final body weight (kg): (**A**,**B**), average daily gain (ADG/kg), and feed efficiency: (**C**,**D**) of dairy heifers of three breed compositions (BCs) at two feeding levels (FLs). Values that differ significantly (*p* < 0.05) are noted with different letters (A, or B). *n* = 72.

The ADG (Figure 1) was affected by FL regardless of BC evaluated, and increased (*p* < 0.001) with the increase in diet supply. The ADG for a high FL was 41.6% greater than the medium FL. There was an interaction between BC and FL (*p* < 0.001) for feed efficiency; in the MFL, Holstein heifers had the highest conversion rate (0.16 kg/kg) compared with Gyr (0.11 kg/kg), while crossbreed HG (0.14 kg/kg) heifers did not differ from the other BCs. The inverse relationship was observed for a high FL; Gyr heifers had 29% greater FE (0.18 kg/kg) compared with Holstein heifers.

**Figure 2:**
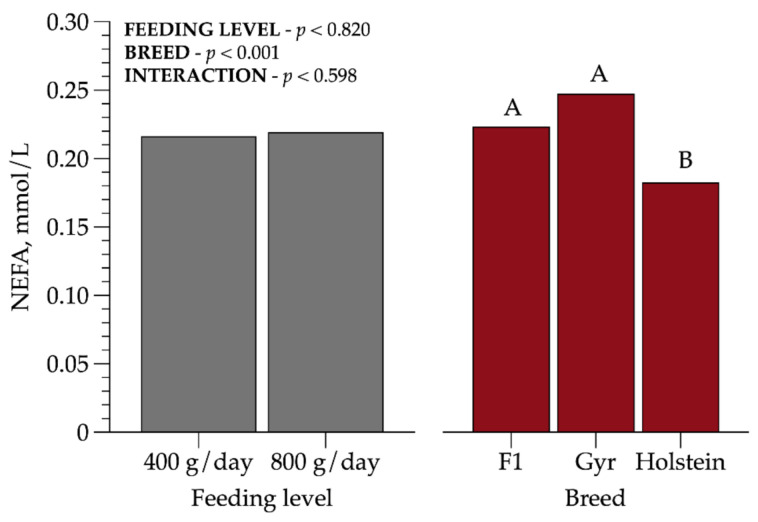
Plasma non-esterified fatty acid (NEFA) concentrations in prepubertal dairy heifers of three breed compositions (BCs) at two feeding levels (FLs). Values that differ significantly (*p* < 0.05) are noted with different letters (A, or B). *n* = 72.

Plasma non-esterified fatty acids concentration was lower (*p* < 0.001) for Holstein heifers (0.18 mmol/L) compared to crossbred HG and Gyr animals. However, none of the heifers were in negative energy balance (Figure 2).

Methane production (g/day; *p* = 0.073; Mcal/day; *p* = 0.037) tended to be lower in Gyr heifers than Holstein and crossbred HG heifers (Table 4). Methane emission increased with increasing FL (*p* = 0.006) and Gyr heifers produced 21% less CH_4_ than Holstein and crossbred heifers. We observed no difference among BC in DMI, which might explain why BC did not influence (*p* > 0.05) CH_4_ yield (CH_4_ per unit of dry matter intake).

**Figure 3:**
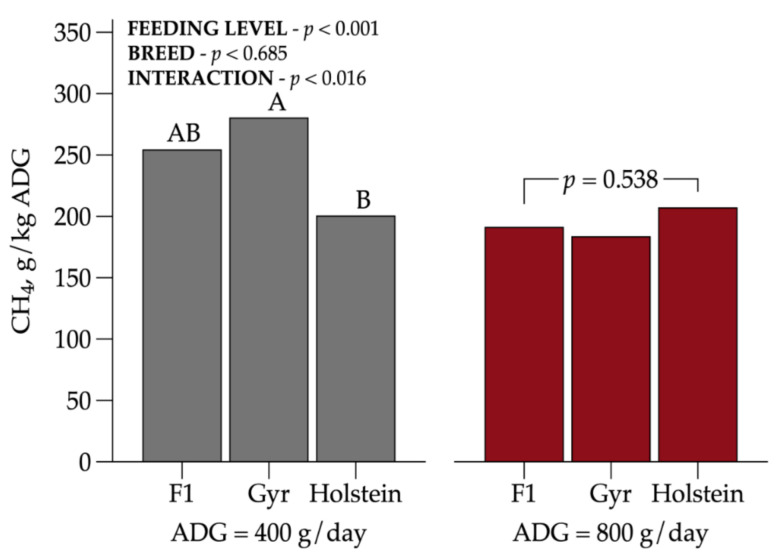
Interaction among dairy heifer breed composition and feeding level on CH_4_ emissions intensity (g/kg ADG). Values that differ significantly (*p* < 0.05) are noted with different letters (A, or B). *n* = 72.

Regarding CH_4_ emissions expressed as g/kg of ADG (Figure 3), there was an interaction between BC and FL (*p* = 0.016). Holstein heifers fed at MPL were more efficient than Gyr animals and obtained lower CH_4_ per ADG. These results suggest that more efficient animals tended to decrease the emissions intensity.

## 4. Discussion

Our hypothesis that BC would influence body weight gain was not confirmed as we did not observe differences in DMI and ME intake among BCs. However, under a higher FL, Gyr and crossbred heifers demonstrated greater feed efficiency than Holstein animals; however, the opposite effect was observed for the MPL.

The prediction of DMI in heifers is a decisive aspect in nutritional programs and an accurate database of dairy heifers DMI of genotypes such as Gyr and HG is not yet available. Pancoti [17] reported a 37% variation in DMI of dairy heifers of different breed compositions raised in tropical conditions. Rennó [18] evaluating Holstein, Zebu, and crossbred (Holstein × Guzerat and Holstein × Gyr) steers also reported differences in intake, whereas Zebu cattle showed lower DMI than Holstein. Comparing those studies to ours, these differences in DMI may be related to the diet composition and experimental conditions. More specifically, in the present study, the performance levels were fixed and greatly affected DMI, which differs from the mentioned studies. Evaluating equations to predict DMI in Holstein and crossbred Holstein × Jersey from four months-of-age until five weeks prepartum, Hoffman [19] found differences when evaluating DMI models and reported that NRC [5] underpredicted DMI of Holstein and crossbred heifers and conversely overpredicted DMI of heavy heifers. Previous studies showed that the level of nutrient intake during the peripubertal period in heifers could have long-lasting effects on productivity, profitability, and reproduction [20,21]. Overall, unbalanced diets may affect the mammary gland development, resulting in fat deposition, decreasing the number of milk secretory cells, and thus negatively affecting future lactation performance [20]. 

Our performance results show that recommendations proposed by NRC [5] for Holstein heifers of 220 kg allowed an ADG of 0.6 kg/d when the animals were fed at MFL and 0.85 kg/d when fed at HFL. Additionally, it should be highlighted that the achieved ADG at a medium FL was 50% higher than the proposed, demonstrating that NRC [5] is overestimating the requirements for animals fed a medium feeding level. Despite the interaction between FL and BC for feed efficiency, Gyr heifers fed at HFL had a greater (0.18 kg/kg) FE than the Holstein breed (0.14 kg/kg) and the crossbred had a similar FE. These BCs are the main basis of the Brazilian dairy herd and research efforts on genetic selection have been showing significant increases in milk production. Due the dual purpose of the Zebu crossbred, they may reach high body condition scores, which can lead to calving problems and future lactation performance [7]. However, our results indicated that Holstein heifers fed at MFL demonstrated a higher FE, which means that the nutrients may be used more efficiently for the basal metabolism and product formation. These data highlighted the importance of meeting nutrient requirements when formulating diets, otherwise, diets may result in negative effects on heifer performance. 

Some authors reported that NEFA is an important indicator of body reserves mobilization [22]. Available literature of dairy cattle suggests NEFA reference values ranging from 0.3–0.7 mmol/L, to prepartum and postpartum, respectively [23]. As we evaluated prepubertal heifers, our findings were below these reference values, but our results support that these heifers did not mobilize adipose tissue to support growth, even in the medium FL. Holstein heifers presented the lowest (*p* < 0.001) value, 0.18 mmol/L when compared to Gyr (0.25 mmol/L) and crossbred HG heifers (0.22 mmol/L).

The main driver of CH_4_ (g/d) emissions in cattle is DMI [24,25], while CH_4_ yield (g/kg DMI) is sometimes decreased with increasing DMI [26]. Gyr heifers emitted 21% less CH_4_ (g/d) than Holstein and crossbred HG heifers. The CH_4_ yield of Holstein and Gyr heifers in the current study were in a similar range as previously reported [24,25], while crossbred HG heifers had a greater CH_4_ yield, but in a similar range as a previous study with Brahman cattle [27]. In the current study, however, DMI was similar among BCs, independent of performance level. Maciel [28] reported no differences between Nellore and crossbred Nellore × Angus fed at feedlot or pasture on CH_4_ yield supporting the findings of the present study, suggesting that BC does not influence CH_4_ yield. The nutrient digestibility of a diet might also affect the amount of CH_4_ emitted, which is dependent on the plane of nutrition [26]. Some studies have shown differences in nutrient digestibility between *Bos taurus*, *Bos indicus*, and crossbred cattle. In general, *Bos indicus* animals have a greater capacity to digest nutrients, especially when fed poor-quality diets rich in fiber [29]. In the present study, however, the digestibility of all nutrients was similar among BCs, except for CP digestibility, which was greater in Gyr and crossbred HG than Holsteins. Despite this difference in CP digestibility, previous studies showed that BC plays a role in the gastrointestinal tract size, so we speculate that Gyr heifers might have a higher retention time, maximizing the digestibility. 

Methane emissions intensity (g/kg ADG) typically decreases with increasing performance [30,31,32]. This corroborates the results from the present study, showing reduced emissions at HFL and confirming that increasing feed intake level leads to a greater reduction in GHG emissions [26]. These data highlighted the importance of a well-managed production system as a strategy of mitigation practices to improve the environmental sustainability in the tropics.

## 5. Conclusions

The present study evaluated performance and methane emission of prepubertal dairy heifers (from 10- until 16-months-old) of three breed compositions fed at two feeding levels. Overall, BC did not influence nutrient intake, however Gyr and crossbred HG heifers had a greater CP digestibility than Holsteins. Confirming our hypothesis, Gyr and HG heifers have greater feed efficiency than Holstein under a high nutrition level. Breed composition does not influence body weight gain of dairy heifers, and Holstein heifers fed the medium feeding level have higher feed efficiency and lower CH_4_ emissions than Gyr. The NRC (2001) overestimates the requirements of animals fed a medium feeding level (400 g/day) as the actual ADG was 50% higher than predicted.

## Figures and Tables

**Table 1:** Ingredients and chemical composition of the experimental diets.

Item	Feeding Level	
	400 g/Day	800 g/Day
Formulation	g/kg DM	
Corn silage	851.0	806.0
Soybean meal	120.0	153.0
Ground corn	0.0	18.0
Urea	11.0	9.0
Ammonium sulfate	5.0	4.0
Mineral mix ^a^	8.0	6.0
Mineral salt	5.0	4.0
Total	1000	1000
Composition	g/kg DM	
Dry matter	373.6	373.2
Crude protein	158.9	145.2
Ether extract	28.3	27.8
Neutral detergent fiber	400.6	404.1
Non-fiber carbohydrates	287.6	300.4
Energy density	Mcal/kg	
Gross energy	4.39	4.43
Metabolizable energy	2.62	2.73

^a^ Guaranteed composition: Ca, 190 g/kg; P, 60 g/kg; Na, 70 g/kg; Mg, 20 g/kg; Co, 15 mg/kg; Cu, 700 mg/kg; Mn, 1.600 mg/kg; Se, 19 mg/kg; Zn, 2.500 mg/kg; I, 40 mg/kg.

**Table 2:** Nutrients intake (kg/d) of dairy heifers of three breed compositions (BC) at two feeding levels.

Item	Feeding Level		Breed			SEM	*p*-Value		
	400	800	F1	GYR	HOL		FL	BC	Int
DM	4.21	5.60	4.92	4.80	5.00	0.587	0.001	0.780	0.157
OM	3.93	5.21	4.58	4.47	4.66	0.537	0.001	0.787	0.153
CP	0.70	0.93	0.83	0.78	0.84	0.120	0.001	0.602	0.269
NDF	1.91	2.44	2.17	2.14	2.21	0.280	0.001	0.831	0.479
EE	0.13	0.17	0.15	0.14	0.15	0.025	0.001	0.442	0.466
NFC	1.18	1.66	1.41	1.41	1.44	0.154	0.001	0.922	0.346
DM/BW	1.67	2.05	1.83	1.88	1.87	0.038	0.001	0.259	0.392
NDF/BW	0.75	0.89	0.81	0.84	0.83	0.030	0.001	0.232	0.476
ME/Mcal, day	11.36	15.20	13.4	13.04	13.41	1.782	0.001	0.913	0.162

DM: dry matter; OM: organic matter; CP: crude protein; NDF: neutral detergent fiber; EE: ether extract; NFC: non-fiber carbohydrate; BW: bodyweight; ME: metabolizable energy. SEM: standard error of the mean; FL: feeding level; BC: breed composition; Int: interaction; *n* = 72.

**Table 3:** Apparent total tract digestibility of dietary nutrients (%) of dairy heifers of three breed compositions (BCs) at two feeding levels.

Item	Feeding Level		Breed			SEM	*p*-Value		
	400	800	F1	GYR	HOL		FL	BC	Int
DM	70.24	70.73	70.40	71.08	69.98	1.032	0.515	0.552	0.751
OM	71.83	72.17	71.90	72.77	71.34	1.019	0.632	0.310	0.695
CP	75.49	75.47	75.69 ^A^	76.7 ^A^	74.05 ^B^	1.708	0.980	0.003	0.795
NDF	57.28	56.7	57.21	58.07	55.7	1.564	0.630	0.283	0.798
EE	83.43	80.5	82.08	82.06	81.74	3.004	0.023	0.969	0.266
NFC	91.62	91.99	92.22	90.57	92.62	2.127	0.546	0.100	0.687

^A,B^ Means within a row with different superscripts are significantly different (*p* < 0.05). DM: dry matter; OM: organic matter; CP: crude protein; NDF: neutral detergent fiber; EE: ether extract; NFC: non-fiber carbohydrate. SEM: standard error of the mean; FL: feeding level; BC: breed composition; Int: interaction; *n* = 72.

**Table 4:** Methane (CH_4_) emission of dairy heifers of three breed compositions (BCs) at two performance levels (PLs).

Item	Feeding Level		Breed			SEM	*p*-Value		
	400	800	F1	GYR	HOL		FL	BC	Int
CH_4_, g/day	125	153	148 ^AB^	117 ^B^	150 ^A^	18.1	0.006	0.073	0.823
CH_4_, g/kg DM	32.6	31.0	32.8	31.2	31.5	2.58	0.376	0.935	0.586
CH_4_, Mcal/day	1.55	2.37	1.97 ^AB^	1.48 ^B^	2.43 ^A^	0.307	0.011	0.037	0.313
CH_4_, g/kg ADG	245	194	223	232	204	24.2	0.001	0.685	0.016
CH_4_, g/BW^0.75^	2.15	2.57	2.21	2.48	2.39	0.195	0.014	0.714	0.651

^A,B^ Means within a row with different superscripts are significantly different (*p* < 0.05). CH_4_: methane; d: day; DM: dry matter; Mcal: mega calories; ADG: average daily gain; BW ^0.75^: metabolic bodyweight. SEM: standard error of the mean; FL: feeding level; BC: breed composition; Int: interaction; *n* = 72.
